# Experiences, views and needs of first-time fathers in pregnancy-related care: a qualitative study in south-East Nigeria

**DOI:** 10.1186/s12884-020-02889-w

**Published:** 2020-04-15

**Authors:** Chiemeka Onyeze-Joe, Isabelle Godin

**Affiliations:** 1 Ecole de Santé Publique, Campus Erasme - CP 596, Route de Lennik, 808, 1070 Bruxelles, Belgium; 2 Centre de Recherche Interdisciplinaire Approches Sociales de la Santé (CRISS), Ecole de Santé Publique, Campus Erasme - CP 596, Route de Lennik, 808, 1070 Bruxelles, Belgium

**Keywords:** Male involvement, First-time fathers, Pregnancy, Qualitative, Nigeria

## Abstract

**Background:**

Given the relevance of paternal involvement in maternal care, there is a need to prepare first-time fathers to participate in pregnancy and childbirth actively. This study explores the experiences and needs of first-time fathers; and how these influences their involvement during pregnancy and childbirth in Nigeria.

**Methods:**

A descriptive qualitative study was conducted. Semi-structured interviews with 50 men recruited from rural and urban workplaces, hospitals, and markets, generated data used to explore the experiences, views and needs of first-time fathers’ in pregnancy-related care in south-east Nigeria. All data were transcribed and analysed using thematic analysis.

**Results:**

Six major themes were identified: gender roles, antenatal involvement, care costs and delivery choices, need to be informed, dealing with emotions, and dealing with the delivery day. The key finding reveals that inexperience and perceptions of gender roles greatly influenced the support provided by first-time fathers to their spouses and the support they received from their social support networks. Two primary needs were identified: need to be informed and the need to know about the cost of care in health settings. First-time fathers acknowledged the role of information on their decision making and final choices.

**Conclusion:**

Findings reveal the influence of gender norms, beliefs, and social support on first-time fathers’ involvement in pregnancy and childbirth. This study also highlights the urgent need to provide informational support for first-time fathers and presents insights into what hospitals can do to achieve this need.

## Background

High maternal mortality remains a reoccurring issue of public health concern in developing countries [1]. Engaging fathers in pregnancy-related care is highly crucial now because they can play a central role that can significantly improve final birth outcomes [2–4]. A growing body of literature promoting the involvement of first-time fathers in maternal care have reported improvements in the utilisation of health services during pregnancy, optimal antenatal care attendance, birth preparedness and improved maternal health outcomes [5–14]. First-time fathers have an important role in providing support for their spouses which creates an all-round sense of security necessary for a successful pregnancy and childbirth experience [15]. Their participation and engagement have been associated with lowered maternal stress levels, lowered occurrences of antenatal and postpartum depression, increased utilisation of maternal health services, readiness for birth complications and positive birth outcomes [10, 12, 14, 16, 17]. First-time fathers are natural providers, who can ensure that the appropriate nutritional and health needs of their spouses are met during pregnancy [7]. Also, with the approaching expected due date, they can provide valuable support, especially in ensuring that important gaps such as those related to finances and transportation are covered [18].

In addition to the role of ensuring maternal wellbeing during pregnancy, father’s involvement is also crucial in healthy infant development [19, 20]. Lamb and colleagues [21] defined the concept of ‘fathers involvement’ as accessibility (being available and present for the child), engagement (contact; bonding and being involved in activities together with the child) and responsibility (participating in decisions that concern the child, e.g. the choice of childcare). Engaging fathers can positively impact the psychological, behavioural, social and cognitive outcomes of the child [20]. Children with highly engaged fathers have been shown to perform better in school [22], relate better socially and are less prone to engaging in risk-related behaviours [23]. However, because of the influence of culture on fathering behaviour, most of these expectations of the ‘involved father’ in the West, are not the same in most patriarchal sociocultural contexts [24].

Transitioning into fatherhood is a significant experience that comes with major biological, psychological and sociocultural changes for many fathers [25, 26]. These changes can impact parental self-efficacy, spousal and extended family relationships and can increase the risk for psychological problems [26–28]. In their systems theory, Cowan and Cowan describe the five dimensions that could represent the transitioning process for most soon-to-be-fathers: his anxieties as a soon-to-be father (the inner life), the desire to be a better father than his father was with him (the quality of relationships in the family of origin), the demands of his job (stress outside the family), the negotiation of new family roles and decisions within his family (the quality of his marriage); and because all these areas of his life are connected, the consequences of a change in one area (the baby) on all other areas of his life [25, 29]. Fathers transitioning to parenthood have been shown to experience elevated levels of stress and other emotions such as worry and fear [18, 30–35]. Although studies explaining paternal psychological health are unpopular in the African context, we know from research, the impact it has on male involvement in pregnancy-related care [28, 32, 36].

The perceptions of male partner involvement during pregnancy differ in societies. In many traditional settings like Nigeria, the perspective of the society regarding all pregnancy and childbirth issues points to the mother of the unborn child [37, 38]. Because women are at the forefront of these issues, the involvement of men is not considered a priority [37, 39]. Maluka and Peneza studied the perceptions of male involvement in pregnancy in Malawi and revealed that the perception of the traditional roles of men hindered their participation in pregnancy and delivery. The study shows that the men had no desire to be more actively involved in antenatal care and delivery beyond their roles as providers [40]. Alternatively, other studies now show a growing willingness in men to be involved in pregnancy and related reproductive health matters [41–43].

Since the twentieth century, societies are experiencing remarkable social and cultural change regarding the roles of fathers and mothers in the family [44]. In the literature, active engagement and support from fathers in western societies have been reported [45–47]. For instance, Pleck et al. describe in their study the evolution of fatherhood in America: from the colonial father to the breadwinner, to the modern involved dad, to the co-parenting father [48]. Within the Saharan African context, some researchers have explored male partner involvement in maternal care [16, 49–53]. However, understanding the needs of first-time fathers at this crucial time remains an understudied area. This study focuses on understanding the needs, experiences and perceptions of pregnancy and childbirth by first-time fathers. Additionally, to understand the impact of their perceptions and needs on their actual involvement in pregnancy and delivery in Nigeria.

### Theoretical framework

In designing this study, the social support theory provided a foundation on which our understanding of the involvement of fathers in pregnancy could be explained. Heaney et al. describe social support as the help provided through social relationships and interactions [54]. Earlier studies have shown how social support networks, comprising of spouses, friends and family members, can be beneficial to people’s health and wellbeing in several contexts [55, 56]. Eldredge et al. [57] identified four main types of social support: emotional support (provision of empathy, love, trust and caring), instrumental support (provision of tangible aid and services), informational support (advice, suggestions) and appraisal (provision of feedback useful for self-re-evaluation and affirmation). In this study context, two aspects of social support were crucial. First, the support provided through the spousal relationship between couples; evident as they help their spouses during pregnancy. Second, first-time fathers’ interactions within their social network as they transitioned into parenthood. This understanding is relevant because first-time fathers in these settings are expected to be in control and to make decisions for the family. Knowing how these interpretations of gender roles, influence the relationship with their spouses, and social support network is also crucial to understanding their involvement and ability to adjust due to need.

## Methods

### Design

A qualitative research design was employed in this study to explore first-time fathers’ experiences, perceptions and needs in pregnancy-related care in Nigeria. The focus of qualitative research is in answering the ‘why’ and ‘how’ questions, enabling in-depth exploration of ‘real-life’ behaviour from the accounts of people. Moreover, different perspectives of reality can be explored to enable a comprehensive understanding of many aspects of human life that quantitative research methods have no answers for [58].

### Ethical consideration

This study received ethical approval from the Ethics Committee of the Hospitals Management Board for Abia State Government. Reference number: HMB/CE/72/002. All participants’ data were renamed with number codes to ensure that their anonymity and confidentiality was protected.

### Settings

This study was performed in both urban and rural settings in south-east Nigeria. In the urban, interviews were conducted in hospitals (one public and three private hospitals) and workplaces (three public offices and one private organisation) located in Umuahia, the capital of Abia State.

Further away from the urban, our studies were performed in two rural communities in Amaba (5112 inhabitants) and Amuzukwu (16,374 inhabitants) both in Abia State. Each village had one health centre with two (Amaba) or three (Amuzukwu) experienced nurses who cared for all the people. These nurses played an essential role in assisting the researcher in recruiting men who accompanied their pregnant spouses for antenatal care. They provided valuable insights on possible locations were eligible participants could be found. Besides these health centres, interviews also occurred in participant’s shops, marketplaces and public locations within the villages.

### Participants and recruitment

Fifty first-time fathers willingly participated out of 70 men contacted to join this study. (see Table 1 above for the sociodemographic characteristics of participants). Twenty fathers were unwilling to participate mainly because of work commitments and possibly because they were not comfortable to discuss a feminine-related topic. Men who were at least 18 years and expecting their first child or experiencing fatherhood for the first time were considered eligible to participate. Interviews were carried out mostly in English and the native language (Igbo). In the initial plan for this study, the researchers projected that all participants needed for this study would be recruited from health settings. However, after 4 weeks of showing up daily in the hospitals, only three participants were recruited from both public and private hospitals. As a result, the researcher changed the recruitment approach to hunting men wherever they were expected to be found, such as in workspaces, marketplaces and pubs. Although most of the first-time fathers were recruited from work settings, obtaining consent from the ‘big bosses’ in the public organisations was challenging without the valuable assistance of a gatekeeper. In all cases, after the first interview occurred, subsequent interview sessions occurred through snowball sampling. For the few recruitments that happened in marketplaces, finding a suitable interview time was challenging because participants could not control the constant interruptions by customers or passersby. The researcher had to adapt by conducting interviews in their shops between 9 am and 11 am when noise and constant human interruptions were less anticipated.

**Table 1:** Sociodemographic characteristics of participants

Total participants	50 men	• Urban • Rural	26 24
Age (23–38 years)	Median (Range)	• Urban • Rural	35 (28–38) 30 (23–35)
Occupation	Public/private workers Private Public	Total • Urban • Rural	30 3 20 7
	Local entrepreneurs		10
	Local farmers		5
	others		5
Education	No education		7
	O′ levels		17
	University education	Total • Urban • Rural	26 22 4
Marital Status	Married	• Urban • Rural	26 19
	Unmarried	• Urban • Rural	- 7
Recruitment	Urban	Workplaces Public hospital Private hospitals Market places	22 1 2 1
	Rural	Health centres Shops/marketplaces Public locations	8 10 6

### Data collection

From the first week of April until the end of July 2017, the first author conducted semi-structured interviews with 50 first-time fathers. Before most interview sessions, a rapport was developed with each participant to create a friendly environment for discussions to occur. During this rapport, the researcher also shared the study objectives and invited them to participate. All willing participants were asked to sign the consent form before interviews commenced. An interview guide created from similar studies served as a guide for the researcher during the interview process [4, 59]. After the opening question, the interview guide was used flexibly. Discussions were covering essential topics such as fathers expected roles during pregnancy and childbirth, antenatal attendance, needs, sources of support, experiences in hospital settings and about personal questions they might have had regarding pregnancy and delivery day. The Interview time varied from one participant to the other depending on the location and the interviewee. On average, interviews lasted for 30 min. However, in some cases, participants (mostly entrepreneurs in the rural communities and junior staff in the public organisations) were willing to be prodded further, which led to more extended interviews of about 40–45 min.

All interviews were audio-recorded with permission. The researcher also took field notes containing observations and other unrecorded discussions.

### Data analysis

All interviews and observational data were transcribed in English by the first author to facilitate familiarity with the data [60]. A local interpreter assisted in the translation of all vernacular words to keep their intended meanings. Thematic analysis utilising the 6-step process outlined by Braun and Clarke was used to explore in-depth the perceptions and needs of first-time fathers directly from the transcribed data [60]. Using an inductive thematic analysis provided an organised and detailed approach, enabling the researchers to identify, analyse and report themes within the data. The first author coded and recoded the data. Initially, eight themes were selected based on patterns identified during the review of the transcripts. Following the revision and redefining of themes by both authors, four fewer reoccurring themes were merged into other active themes. After several backs and forth reviews, the authors agreed on the six final themes presented in the findings. To further validate our data, the results were discussed with the first author’s academic team to ascertain whether the six themes reflected the objectives of the study.

## Results

The six themes were: (I) gender roles, (II) antenatal involvement, (III) care costs and delivery choices, (IV) need to be informed, (V) dealing with emotions, and (VI) dealing with the delivery day. Themes (V) (dealing with emotions) and (VI) (dealing with the delivery day) have two sub-themes each, that highlight first-time father’s accounts regarding their emotions and perceptions about the delivery day.

### Gender roles

Overall, the first-time fathers emphasised the primary role expected of the traditional *Igbo man,* which is to be the provider of his family. As illustrated by an expectant father,

“I do my part as all men should, I give her money for food, I provide money to go to the health centre”. (H-SS 41)

Here, the first-time fathers reported feeling satisfied that their wives are well taken care of because they were providing financially. Some first-time fathers strongly affirmed that both men and women have culturally defined roles that remain the same, whether a woman is pregnant or not. One male partner expressed this by saying: “In the family, everybody has his or her role. I do not expect my wife to bring money; it is not her duty to wash the car or change the light bulb. It is the duty of the man; when I come home, I expect her to go and prepare food”.

In contrast, some first-time fathers willingly shared domestic responsibilities with their spouses within the confines of their homes alone. These first-time fathers were willing to take up extra duties to make their spouses feel loved and happy in pregnancy. Less common were accounts of first-time fathers ready to be more involved in hospital settings despite the risk of ridicule by their peers. This category of fathers admitted to being more concerned with the wellbeing of their spouses even if they were perceived as ‘weak men’ in the eyes of society.

### Antenatal involvement

Antenatal involvement here refers to men accompanying their spouses to health settings for prenatal appointments. One reoccurring discussion with first-time fathers during the interviews addressed the issue of antenatal attendance. Evident in the male partner’s descriptions were conflicting views of how men could be involved in prenatal care. There were men in urban settings, who spoke about their willingness to participate occasionally in hospital settings but could not because of their jobs. Several of such first-time fathers acknowledged dropping their spouses off for their antenatal appointments and often calling to check up on their spouses. One male partner said that he made up for his absence during the day by coming home early and taking exercise walks with his wife most evenings after work. In rural settings, more first-time fathers occasionally attended antenatal care appointments with their spouses than in urban environments. Most of these men were local transporters, owners of local businesses or farmers who acknowledged their ability to attend these sessions because they had flexible work hours. Also, most of the men in this category, acknowledged several motivations to participate in antenatal sessions which were mainly to know about pregnancy and to make their spouses happy.

“We visit the clinic together because we do get information about a lot of things. Being here with her in the hospital does not mean that I do not have work to do now, but I want to make her feel happy. Seeing me around her makes her feel like she is not the only one carrying the pregnancy” (AM H1).

In contrast, some first-time fathers believed that antenatal care attendance was strictly for the pregnant woman. Among these, where first-time fathers who provided money for their spouses to register in a health setting and let their spouses manage the pregnancy with the help of other experienced women. One participant’s response regarding this was:

“My wife is a matured person in mind; she handled the pregnancy well. She had adults around her, her friends, our mothers and her sisters. She did all the consultations by herself. Sometimes, she told me about them, only if it had to do with money” (H-FMC 9).

### Care costs and delivery choices

Occasionally first-time fathers admitted their concerns over costs, especially those associated with delivery. There was a common desire among first-time fathers to know about the cost of care for vaginal and caesarean births in both public and private hospital settings. The knowledge about the financial cost of care was necessary for most to know their options and to compare prices, before deciding on which health setting to choose for delivery. In most cases, most first-time fathers admitted that the availability of funds influenced the choice of a delivery place.

“You know money plays a huge role in deciding where your wife is going to deliver” (FMC,001).

“As I told you before, I had lost my job, and we could not even afford to go to a hospital…. we had this maternity close to our home. That is where my wife registered. The midwife understood our financial condition and took care of my wife properly from pregnancy until she gave birth” (H-FMC-002).

On the other hand, some first-time fathers had no worries about funding care costs. Among these were civil servants working with the government who had opted for the national health insurance scheme (NHIS). These first-time fathers acknowledged the freedom to choose quality safe services without worrying over costs of care on the day of delivery. Besides**,** an uncommon conversation with a participant revealed a preference for an affordable health facility in a foreign country because he desired a safe birth and an opportunity to give his unborn child a good start in life. The participant stated**,** “When my wife’s pregnancy was confirmed, I started making plans to fly her abroad. It is a costly venture, but you are sure she is in good hand. Later, when the child grows up, he can attend a proper school there too” (PP-003).

### Need to be informed

Familiar across the first-time fathers’ accounts was the feeling of ignorance concerning pregnancy and childbirth because they lacked experience. The first-time fathers admitted to tackling their inexperiences in a ‘*manly*’ way, by seeking the opinions of people they trust or sending their spouses with questions to the health centres. Often this form of support came from a few close friends and neighbours, family members, experienced mothers, experienced colleagues, and most especially from spiritual leaders. Some first-time fathers shared stories from the experiences of others and related these stories to their situations.

“I heard all sorts of stories about delivery. You have to pay for everything before they even touch your pregnant wife, no one will attend to you without money. I just knew I had to start saving money” (H-SS-015).

“You know my brother’s experience taught me a lot of things about pregnancy. You cannot give a pregnant woman medication no matter what. Now, if she complains about something as little as fever, we go to the hospital” (HSS-039).

While some first-time fathers acknowledged their principal support from their relationships and networks; others preferred not to discuss this with people in their support networks. They admitted to reading books, surfing the internet and speaking only with health professionals for their information needs. Among these were first-time fathers who desired to keep the pregnancy news a secret. One male partner said,

“You know that this Igbo culture has made it that you don’t go about telling people that your wife is pregnant. For me, too, I didn’t tell people. I was building information from the internet and books, and I will probably go to the hospital only when there is an emergency or sickness” (H-FMC-005).

### Dealing with emotions

#### V(i) feeling overwhelmed, worried and scared

First-time fathers voiced their feelings on how the experiences of their wives during the pregnancy and delivery affected them. Some comments such as “the feelings this process created in me was overwhelming”, “I became very worried”, “I was so scared” reflected this. One participant feared so much for the life of his pregnant wife that he took his wife to see a doctor for an abortion.

“Honestly, I did not know that a pregnant woman can be sick and vomit continuously like that for days. After preparing delicious foods, she eats and then vomits everything. She sometimes would vomit like she is going to die. One day I was so afraid that I told her let’s go to the hospital and terminate the baby. I told the doctor clearly that I wanted an abortion. The doctor smiled and started educating me” (H-SS-011).

Also, some first-time fathers spoke about the unpleasantness of their spouse’s changing food cravings. In his own words, a male partner said:

“Managing her food cravings was a horrible experience for me... One late evening, she demanded Pepsi, on arrival, she demanded malt. I had to go out again to buy that. Sometimes after cooking, she will not feel like eating anymore; she will ask me to find one roadside food for her” (H-FMC-005).

Then, some first-time fathers felt differently about being around their pregnant spouses. These fathers described the emotional mood swings their spouses experienced and how irritable that made them feel. One partner admitted that “when a woman feels this way, she should be left alone”. He said that he often chose to hang out with his friends to keep his sanity than stay home with his spouse at such times.

#### V(ii) being strong for their spouses

‘Being strong for their spouses’ refers to first-time fathers need to be the strong and supportive one despite their feelings. Several first-time fathers relayed peculiar situations where they had to be in control in other for their spouses to feel secure. While these men had their own emotions to deal with, they admitted to masking these emotions in a masculine appearance of strength and courage as is expected of men in these settings.

“She made me worry a lot when I look at her, I say every woman can give birth, but I don’t know if she can because she was so fragile in my eyes.…. I had to be the strong one” (H-SS 12).

Some first-time fathers talked about some discussions they had with other men in their networks, which allowed them to see the experiences of other men in the light of their daily struggles. For many, having these types of discussions in their workplace or among ‘hangout’ associates, prepared them to tackle their problems.

### Dealing with delivery day

#### V(i) to be present or not

During the interviews, first-time fathers shared their thoughts about their experiences on the day of delivery. It was clear from their responses that being present in the delivery room was not a popular opinion. Most first-time fathers believed that the maternity ward is a strictly feminine environment, and some others were afraid of witnessing the delivery experience. A few first-time fathers acknowledged their willingness to be present but could not because the choice to be involved was not given to them by the hospitals. Here are some comments

“they will not allow you in; they told me to stay outside” (H-LGA-1).

“From my experience, I think all men should be in there. But you know all men will not have the mind to be in the delivery room, for those who can withstand these things, I will encourage them to attend” (H-FMC-6).

“I did not want to go into that room at all. I prefer to stay outside and hear the news from outside. I don’t think it will make any difference if I am there or not. Some friends told me that if I go in there, I should not be surprised if my woman starts to beat me and kick me because I am the cause of her pain” (H-FMC-4).

Although absent in health settings; some first-time fathers mentioned how they ensured that an experienced woman (examples: sisters, mothers or mothers in law) was always present. Her role was to assist their spouses during delivery and afterwards for at least 3 weeks. One participant stated,

“Towards the delivery period, all I need to do is to arrange my mother or hers to come and hang around” (H-FMC-005).

Other supportive activities mentioned by the first-time fathers during delivery were offering spiritual support, running errands for their spouses, and signing relevant documents when needed.

#### V (ii) social support in health settings

The accounts of first-time fathers, especially in urban locations, indicated how the presence of a friend working in the hospital could facilitate access to care, especially if labour occurred at night. These first-time fathers shared instances of pregnant women being neglected or maltreated in the hospital in the absence of this type of support.

“My friend had a bad experience at the public hospital. He got there past 10 pm in the night; the doctors had deserted, the nurses were busy chatting and gossiping, his wife was in labour. He caused a scene before they could attend to him. He told me, it had to take him calling a doctor he knew before he could receive the appropriate assistance that night. She nearly lost her life due to their negligence” (HSS-015).

## Discussion

This study explored the experiences, views and needs of first-time fathers regarding pregnancy and delivery in south-east Nigeria.

Our findings show that the perception of men as providers strongly dominated the action of first-time fathers in this cultural setting. In Chinua Achebe’s ‘*Things fall apart’*, the *Igbo man* is culturally depicted as industrious, and the sole provider and protector of his home [61]. It is expected of him to be in control and to dominate his immediate territory (his family). Through socialisation, gender norms are internalised in the mind of the ‘*traditional Igbo’* man from childhood. These internalised norms continue to shape the perceptions of his roles in adulthood and subsequently influences his behaviour through life [62]. From this study, it is evident that the primary form of support expected of the man during pregnancy and childbirth is to provide. Achieving this was expressed strongly with satisfaction and pride. Other supportive roles (such as providing emotional, spiritual or domestic support) were regarded as non-obligatory and secondary responsibilities: roles family and friends could perform in their stead. Our findings agree with Matseke et al. [4]. This study revealed that men who defined partner support as limited to giving financial help did not endorse the view of carrying out physical tasks for pregnant women [4]. First-time fathers aspired to be that ‘good father’ who provides; in addition to the responsibility of providing practical and emotional support to their partners [63].

The literature supports first-time fathers involvement in antenatal sessions because they are better informed and prepared for their transition into parenthood [46]. On the one hand, very few first-time fathers accompanied their spouses to antenatal sessions or wished to be present during delivery. Most men believed that it is not expected of them to be present with their spouses when they should be at work. However, there were men in this study who were willing to attend antenatal sessions and support their spouses during delivery. Unfortunately, most health settings in our study were more equipped to attend to women exclusively, creating an uncomfortable presence for any man who dared to be present. Similar studies have also revealed how first-time fathers often felt excluded in hospitals, too [29, 64]. Therefore health settings must revise their strategies to create alternative services for men, that is inclusive. These strategies should operate in a culturally acceptable and friendly framework to encourage their involvement in maternal care. For instance, Robertson [65], revealed that organising male-specific sessions might be a great way to inform men because they would tackle topics relevant to them as men. In this way, they can support each other in common areas of interest [66].

This study did not explore the perspectives of pregnant women regarding antenatal attendance with their husbands. If pregnant women do not desire their husbands to accompany them for prenatal care, it might be a challenge to persuade men themselves. Some studies have shown that pregnant women can discourage their spouses towards antenatal attendance due to reasons deeply embedded in the cultural perceptions of the people [10]. A study in Malawi revealed how important it is for the pregnant woman to see reasons why fathers must be involved in maternal care [10]. There is a possibility that informed women will coerce their spouses to be involved because they understand the impact on their overall health and that of their unborn child [10].

Although these men adhere naturally to gender norms, our study reveals a willingness in some first-time fathers to go beyond the norm and to carry out other feminine tasks during pregnancy. For instance, some first-time fathers preferred to be ridiculed by their peers or perceived as ‘weak’ in the eyes of society for taking up ‘female roles’ or being present with their spouses in hospital settings. This stance speaks of the willingness of some first-time fathers to be more flexible and stand on their convictions despite the perceived shame. Breaking out of culturally shaped modes of gender roles are strong indications of a changing era. The literature acknowledges this changing era and describes it as one marked with evolutions and social changes in the cultural way of life of people globally. In their studies, Cabrera et al. revealed that one of the prominent social trends that resonate with the twenty-first century is the increasing involvement of fathers in family life [44]. These trends are revolutionising long-standing beliefs and redefining our perception of gender roles within the family [67]. With the increasing preference for western lifestyle in these settings, it is anticipated that the era of uninvolved fathers will gradually disappear, ushering in a new era of more involved men.

The literature has shown that fathers involved during pregnancy and childbirth are more likely to develop their parental identity early enough than fathers who do not [46]. Although first-time fathers in this study eagerly anticipated fatherhood with joy, most acknowledged how unprepared they were in handling the experiences they encountered in the process. It is common for men not to show ‘weak emotions’ in these settings. Therefore, first-time fathers in this study preferred to maintain the outward look of strength for their spouses and tackle their emotional challenges quietly. This finding is consistent with a study among Danish fathers [68]. This study reports how the fathers would instead take time out of the hospital than show any feelings that reveal they are not in control of their emotions [68]. While the men did not admit to seeking information specifically for their emotional needs, they spoke about the influence of their social support networks during some difficult times. Some first-time fathers talked about the emotional and informational support they received from people such as their religious leaders, family members and experienced friends. Although these forms of support occurred the most through face to face interactions with people in their support networks, this study observed a growing utilisation of online resources by first-time fathers. More research is needed to explore this growing trend.

Furthermore, the first-time fathers acknowledged the role of the information they received especially on the cost of care in their decision making and final delivery choices afterwards. Health care costs in Nigeria are mostly out of pocket. Hence most fathers would prefer to scope for affordable services before their final decisions are made [69]. This might be the reason why fathers with low income, were more likely to opt for riskier options (e.g. a traditional birth attendant) over a public/private hospital, because of costs. Similar findings were reported in a study in Kenya, in which fathers with low income were also concerned about costs [70]. These men were discouraged by the additional costs of transportation to the clinics and the unexpected costs associated with antenatal and postnatal care services [70].

## Conclusion

From these findings, it is evident that the indigenous culture and the perception of gender roles dominate the actions of first-time fathers regarding their involvement in pregnancy and childbirth. Regrettably, most first-time fathers are inexperienced, often misinformed and the majority still think they are only required to provide financially at this crucial time. Our study reveals the critical need to introduce information sessions for first-time fathers and to promote health schemes that encourage their involvement in maternal and child health. Our study also highlights an absent discussion in Nigeria’s health community about the emotional and mental health of first-time fathers.

### Recommendations

Considering the strong influence of patriarchy, a positive and impactful strategy will require a culturally specific health intervention in teamwork with local leaders, men and local health institutions. Working with local leaders as change agents can inspire first-time fathers in this setting [71]. Our study also recommends a man to man strategy: using male-only information sessions to motivate more men to attend. This approach will also provide an opportunity for men to learn from each other’s experiences as well [71, 72].

A growing number of educated men in this study utilised online resources for their questions regarding pregnancy. It is vital to explore this health information-seeking behaviour in first-time fathers and to discover their areas of interest and need. Our study recommends creating online social communities, which have the potential of reaching a wider audience without the limitations of time and space. These online platforms can be adapted to be interactive and offer emotional and instrumental support as well. Additionally, local health organisations can utilise social media platforms to boost first-time fathers’ engagement, disseminate information and create awareness.

### Implications for health services and researchers

This study’s results have implications for health services. Firstly, including husbands as early as possible in prenatal care can be a turning point in getting more men involved during pregnancy, childbirth and in the entire process of infant development. To encourage more men to attend, we propose at least one male-only informational meeting, which should occur in hospital settings. To achieve this goal, health professionals can give out invitations to pregnant women attending antenatal check-ups for their husbands. Consequently, health professionals will have the opportunity to educate the men and gradually reduce the cultural awkwardness surrounding male involvement in pregnancy-related matters in hospital settings.

Secondly, creating online support services can be highly relevant to reach the increasing population of first-time fathers relying on online platforms for informational support. In addition to this, considering that most men have mobile devices, text messaging can be utilised in disseminating information to men who subscribe to these tips [73].

For researchers, our study calls for more studies focusing exclusively on fathers transitioning into parenthood in sub-Saharan Africa. Such research may explore the psychological and emotional health of first-time fathers and the impact on their relationships, lifestyle and involvement in infant development.

### Study limitations

First, most of the men interviewed in this study had primary education (at least O levels). It is possible that conducting interviews with uneducated first-time fathers would provide additional or different perspectives on men’s experiences and needs in pregnancy and delivery. Secondly, to reflect the impact of gender roles on male involvement, other opinions from the spouses might have provided a richer understanding of the expected roles of men during pregnancy and delivery in these settings. Besides, within this setting, we think the men will say anything to project their being in control. Hence, a different perspective from their wives would have proven if their responses were accurate or not.

Further research to include mothers, wives and the family may yield a richer understanding of how family, beliefs, education influence first-time fathers’ participation in pregnancy and childbirth.

Finally, although the researcher was flexible, it was challenging to find first-time fathers willing to be interviewed in hospital settings and the marketplace.

## Data Availability

The datasets generated and analysed during the current study are not publicly available because releasing such datasets might compromise participants privacy and anonymity. Hence, data will only be made available from the corresponding author on reasonable request.

## Abbreviations

FMC: Federal medical Centre
NHIS: National Health Insurance Scheme
